# Case 3/2017 - High Origin of the Right Coronary Artery at the Sinotubular Junction, in a 14-Year-Old Teenager, in Diagnostic Imaging Diversity

**DOI:** 10.5935/abc.20170048

**Published:** 2017-04

**Authors:** Edmar Atik, Gabriela Leal

**Affiliations:** Clínica Dr. Edmar Atik, São Paulo, SP - Brazil

**Keywords:** Myocardial Ischemia, Coronary Vessels, Echocardiography, Doppler, Sinus of Valsalva, High origin of right coronary artery

## Clinical data

He reports that, six months ago, after discreet exercise (having run about 500 meters) he felt tiredness and dizziness, malaise and skin paleness. Repeated migraines accompany the clinical status. Recent bi-Doppler echocardiography revealed the high origin of the right coronary artery at the sinotubular junction. There was no morbid past of importance.

Physical examination: eupneic, acyanotic, normal pulses. Weight: 66 Kgs, Height: 169 cm, BP: 110/65 mm Hg, HR: 57 bpm, O2 saturation = 96%. The aorta was not palpated at the suprasternal notch.

In the precordium, without systolic impulses, the *ictus cordis* was not palpated. Cardiac sounds were normal and heart murmur was not heard. The liver was not palpated.

### Additional Examinations

**Electrocardiogram** showed sinus rhythm and signs of electrical normality. The duration of the QRS complex was 0.092` with morphology rS in V1 and qRs in V6, with negative T wave in V1. AP: + 40°, AQRS: + 75°, AT: + 25°.

**Figure1:**
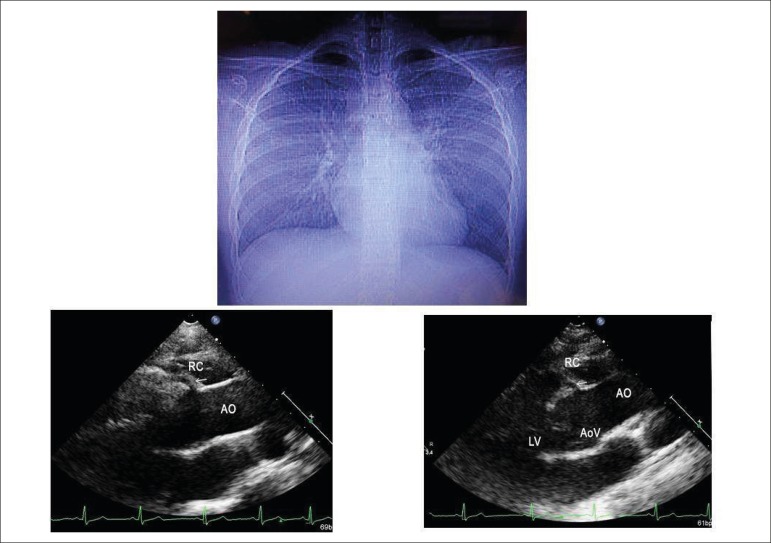
Chest X-ray shows normal cardiac area and pulmonary vascular markings and parasternal long-axis view echocardiogram clearly points to the high origin of the right coronary artery at the sinotubular junction. Ao: aorta; RC: right coronary; AoV: aortic valve, LV: left ventricle.

**Chest X-ray** shows normal cardiac area and pulmonary vascular markings (Figure 1).

**Image exams: Bi-Doppler echocardiogram** showed cardiac cavities of normal size and function. The dimensions were, in Ao = 24; RA = 28; RV = 20; LV = 49; ventricular septum and posterior wall of LV = 7; Ventricular ejection fraction = 65%. The right coronary artery in the parasternal long axis view showed high origin at the sinotubular junction in clear oblique orientation between the aorta and the pulmonary trunk (Figure 1).

**Tilt-test** showed no significant changes in blood pressure (103/66 to 104/65 mmHg) and heart rate (from 77 to 94 bpm).

**Dynamic 24-hour electrocardiogram (Holter)**: showed no changes in heart rhythm and / or waves, complex and electrical segments.

**Figure 2:**
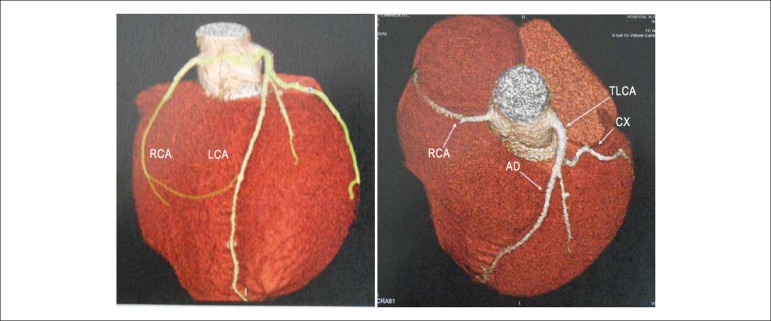
Angiotomographies of the coronary arteries, in two distinct views, show normal origins and at the same height of both. AD: anterior descending; Cx: circunflex artery; LCA: left coronary artery; RCA: right coronary artery; TLCA: trunk of left coronary artery.

**Angiotomography of the coronary arteries** revealed normal and usual origin of them in the different sinuses of Valsalva and both at the same height (Figure 2).

**Myocardial scintigraphy with physical exertion** did not reveal any myocardial ischemic changes.

### Clinical diagnosis

High origin of the right coronary artery at the right sinotubular junction by echocardiogram, in a teenager with nonspecific symptoms, not confirmed by angiographic study.

### Clinical reasoning

The clinical elements were compatible with cardiovascular normality with nonspecific symptoms. The finding of an echocardiographic examination with a high origin of the right coronary artery led to a more accurate investigation of the existence of myocardial ischemia, not evidenced in myocardial scintigraphy and angiotomography.

### Differential diagnosis

The anatomical and ischemic findings of the heart in teenagers and young adults occur in several other situations, such as the origin of coronary artery from the contralateral sinus of Valsalva (most common coronary anomaly of all), as well as in hypoplastic coronary arteries, in stenosis in the coronary ostium-*slitlike shape*, in aortic and also interarterial intramural tracts, between the aorta and the pulmonary artery, besides the acute angle formed between the coronary and the aorta in descending straight path, and finally in the early atherosclerotic disease. All these conditions are well recognized as causing ischemia and sudden death after physical exertion, and with prodromes of palpitations, syncope or precordial pain.

### Conduct

As the findings of the echocardiogram did not find any correspondence with the normal images revealed by angiotomography and consolidated by the functional study of myocardial scintigraphy, there was the recommendation of periodic follow-up without limitation of the usual activities. It was difficult to explain the echocardiographic finding that was characterized by a diagnostic error of this coronary anomaly. Hence, the need for an ever more accurate assessment in order to confirm the diagnosis, having as support the analysis of all the elements that were exhaustively pursued in the case in question.

## Comments

High origin of the coronary artery is very rare (0.1% of the right coronary artery and 0.7% of the left of all coronary abnormalities) and in the reported cases, associated ischemia is systematically correlated with the presence of other additional abnormalities. Therefore, it is difficult to implicate high coronary origin as a cause of ischemic event and hence as being definitely pathological. It may predispose to myocardial ischemic alterations in the presence of associated abnormalities, such as in a single coronary artery, in a vertical and oblique path between the aorta and the pulmonary artery, allowing obstruction by compression and with consequent reduction of coronary flow, as well as a narrow coronary ostium^1^ and intramural aortic. Thus, in findings of high origin of the coronary artery we should immediately eliminate the presence of myocardial ischemia due to decreased ostium (50% of the diameter), interarterial pathway or presence of another abnormality.However, there are authors who believe in the hypothesis of myocardial ischemia only because of the high coronary origin due to the reduction of the diastolic filling of the coronary, being able in addition to theoretically cause chronic myocardial damage with consequences in adulthood. Thus, the question is if the high origin of the coronary artery constitutes a benign or malignant anomaly.^2,3^
